# 3,5-Bis(4-methyl­phen­yl)-1-phenyl-4,5-dihydro-1*H*-pyrazole

**DOI:** 10.1107/S1600536811011494

**Published:** 2011-03-31

**Authors:** Ray J. Butcher, Mehmet Akkurt, S. Samshuddin, B. Narayana, H. S. Yathirajan

**Affiliations:** a Department of Chemistry, Howard University, 525 College Street NW, Washington, DC 20059, USA; b Department of Physics, Faculty of Sciences, Erciyes University, 38039 Kayseri, Turkey; c Department of Studies in Chemistry, Mangalore University, Mangalagangotri 574 199, India; d Department of Studies in Chemistry, University of Mysore, Manasagangotri, Mysore 570 006, India

## Abstract

In the title compound, C_23_H_22_N_2_, the dihedral angle between the methyl­benzene groups is 77.62 (6)°, and the dihedral angle between the envelope-shaped pyrazole ring [in which one C atom displaced by 0.109 (1) Å from the mean plane of the other four atoms] and the phenyl ring is 17.57 (7)°. The dihedral angles between the phenyl ring and the two methyl­benzene rings are 13.24 (6) and 81.02 (7)°. In the crystal, weak C—H⋯π inter­actions link the mol­ecules.

## Related literature

For related structures and background references, see: Jasinski *et al.* (2010) ▶; Samshuddin *et al.* (2010 ▶).
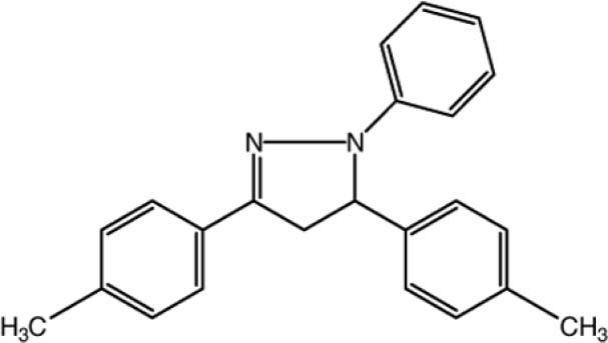


## Experimental

### 

#### Crystal data

C_23_H_22_N_2_
*M* *_r_* = 326.43
Monoclinic, 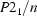

*a* = 5.8113 (3) Å
*b* = 10.6959 (5) Å
*c* = 28.4455 (13) Å
β = 94.983 (4)°
*V* = 1761.41 (15) Å^3^
*Z* = 4
Cu *K*α radiation
μ = 0.55 mm^−1^
*T* = 123 K
0.53 × 0.11 × 0.07 mm

#### Data collection

Oxford Diffraction Xcalibur Ruby Gemini CCD diffractometer
Absorption correction: multi-scan (*CrysAlis PRO*; Oxford Diffraction, 2007 ▶) *T*_min_ = 0.736, *T*_max_ = 1.000
12872 measured reflections
3615 independent reflections
3096 reflections with *I* > 2σ(*I*)
*R*_int_ = 0.033

#### Refinement

*R*[*F* ^2^ > 2σ(*F* ^2^)] = 0.043
*wR*(*F* ^2^) = 0.119
*S* = 1.03
3615 reflections
228 parameters
H-atom parameters constrained
Δρ_max_ = 0.31 e Å^−3^
Δρ_min_ = −0.21 e Å^−3^

### 

Data collection: *CrysAlis PRO* (Oxford Diffraction, 2007 ▶); cell refinement: *CrysAlis PRO*; data reduction: *CrysAlis RED* (Oxford Diffraction, 2007 ▶); program(s) used to solve structure: *SHELXS97* (Sheldrick, 2008 ▶); program(s) used to refine structure: *SHELXL97* (Sheldrick, 2008 ▶); molecular graphics: *ORTEP-3* (Farrugia, 1997 ▶); software used to prepare material for publication: *WinGX* (Farrugia, 1999 ▶) and *PLATON* (Spek, 2009 ▶).

## Supplementary Material

Crystal structure: contains datablocks global, I. DOI: 10.1107/S1600536811011494/hb5825sup1.cif

Structure factors: contains datablocks I. DOI: 10.1107/S1600536811011494/hb5825Isup2.hkl

Additional supplementary materials:  crystallographic information; 3D view; checkCIF report

## Figures and Tables

**Table 1:** Hydrogen-bond geometry (Å, °) *Cg*2, *Cg*3 and *Cg*4 are the centroids of the C4–C9, C10–C15 and C17–C22 rings, respectively.

*D*—H⋯*A*	*D*—H	H⋯*A*	*D*⋯*A*	*D*—H⋯*A*
C2—H2*B*⋯*Cg*3^i^	0.99	2.74	3.5766 (13)	142
C12—H12*A*⋯*Cg*2^ii^	0.95	2.69	3.5485 (15)	150
C16—H16*C*⋯*Cg*4^iii^	0.98	2.81	3.6144 (17)	140
C23—H23*B*⋯*Cg*4^iv^	0.98	2.77	3.5742 (16)	140

Symmetry codes: (i) 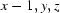; (ii) 
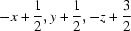
; (iii) 
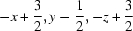
; (iv) 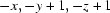.

**Fig. 1.**
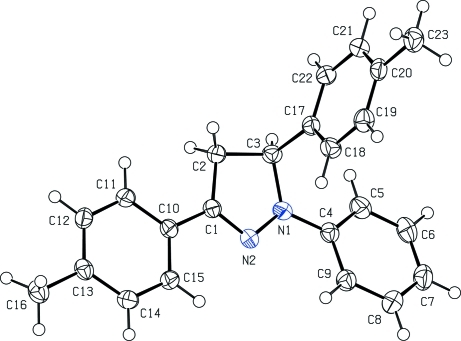
Molecular structure of the title compound showing displacement ellipsoids for non-H atoms drawn at the 50% probability level.

**Fig. 2.**
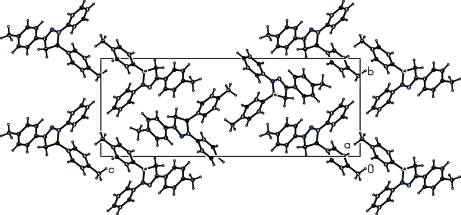
Packing diagram of the title compound viewed down the a axis.

## supplementary crystallographic information

### Comment

In continuation of our work on pyrazoline derivatives Samshuddin *et al.*, 2010, Jasinski *et al.*, 2010), we now describe the synthesis and structure of the title compound, (I).

The title compound (I) contains two methylbenzene groups and a phenyl ring attached to an envelope configured pyrazole ring (Fig. 1). The dihedral angle between the two methylbenzene groups is 77.62 (6)° and the dihedral angle between the pyrazole and phenyl rings is 17.57 (7)°. Also, the dihedral angles between the phenyl ring and the two methyl-substituted phenyl groups are 13.24 (6) and 81.02 (7)°, respectively. Four C–H···π interactions (Table 1) contribute to the stability of the crystal structure (Fig. 2).

### Experimental

A mixture of (2*E*)-1,3-bis(4-methylphenyl)prop-2-en-1-one (2.36 g, 0.01 mol) and phenyl hydrazine (1.08 g, 0.01 mol) in 50 ml glacial acetic acid was refluxed for 6 h. The reaction mixture was cooled and poured into 50 ml ice-cold water. The precipitate was collected by filtration and purified by recrystallization from ethanol. Yellow needles of (I) were grown from acetonitrile by slow evaporation (m. p.: 412–414 K, yield: 78%).

### Refinement

All H atoms were placed in their calculated positions (methyl C—H = 0.98 Å, methylene C—H = 0.99 Å, methine C—H = 1.00 Å and aromatic C—H = 0.95 Å) and refined using a riding model. Isotropic displacement parameters for these atoms were set to 1.2 (or 1.5 for the methyl group) times the *U*_eq_ of the parent atom.

### Crystal data

**Table:**

C_23_H_22_N_2_	*F*(000) = 696
*M**_r_* = 326.43	*D*_x_ = 1.231 Mg m^−^^3^
Monoclinic, *P*2_1_/*n*	Cu *K*α radiation, λ = 1.54178 Å
Hall symbol: -P 2yn	Cell parameters from 6406 reflections
*a* = 5.8113 (3) Å	θ = 4.4–75.5°
*b* = 10.6959 (5) Å	µ = 0.55 mm^−^^1^
*c* = 28.4455 (13) Å	*T* = 123 K
β = 94.983 (4)°	Needle, yellow
*V* = 1761.41 (15) Å^3^	0.53 × 0.11 × 0.07 mm
*Z* = 4	

### Data collection

**Table:**

Oxford Diffraction Xcalibur Ruby Gemini CCD diffractometer	3615 independent reflections
Radiation source: Enhance (Cu) X-ray Source	3096 reflections with *I* > 2σ(*I*)
graphite	*R*_int_ = 0.033
Detector resolution: 10.5081 pixels mm^-1^	θ_max_ = 75.7°, θ_min_ = 4.4°
ω scans	*h* = −7→6
Absorption correction: multi-scan (*CrysAlis PRO*;Oxford Diffraction, 2007)	*k* = −13→13
*T*_min_ = 0.736, *T*_max_ = 1.000	*l* = −34→35
12872 measured reflections	

### Refinement

**Table:**

Refinement on *F*^2^	Primary atom site location: structure-invariant direct methods
Least-squares matrix: full	Secondary atom site location: difference Fourier map
*R*[*F*^2^ > 2σ(*F*^2^)] = 0.043	Hydrogen site location: inferred from neighbouring sites
*wR*(*F*^2^) = 0.119	H-atom parameters constrained
*S* = 1.03	*w* = 1/[σ^2^(*F*_o_^2^) + (0.0644*P*)^2^ + 0.4363*P*] where *P* = (*F*_o_^2^ + 2*F*_c_^2^)/3
3615 reflections	(Δ/σ)_max_ = 0.001
228 parameters	Δρ_max_ = 0.31 e Å^−^^3^
0 restraints	Δρ_min_ = −0.21 e Å^−^^3^

### Special details

**Table:**

Geometry. Bond distances, angles *etc*. have been calculated using the rounded fractional coordinates. All su's are estimated from the variances of the (full) variance-covariance matrix. The cell e.s.d.'s are taken into account in the estimation of distances, angles and torsion angles
Refinement. Refinement on *F*^2^ for ALL reflections except those flagged by the user for potential systematic errors. Weighted *R*-factors *wR* and all goodnesses of fit *S* are based on *F*^2^, conventional *R*-factors *R* are based on *F*, with *F* set to zero for negative *F*^2^. The observed criterion of *F*^2^ > σ(*F*^2^) is used only for calculating -*R*-factor-obs *etc*. and is not relevant to the choice of reflections for refinement. *R*-factors based on *F*^2^ are statistically about twice as large as those based on *F*, and *R*-factors based on ALL data will be even larger.

### Fractional atomic coordinates and isotropic or equivalent isotropic displacement parameters (Å^2^)

**Table:**

	*x*	*y*	*z*	*U*_iso_*/*U*_eq_	
N1	0.03560 (19)	0.23302 (10)	0.66268 (4)	0.0274 (3)	
N2	0.24215 (19)	0.20597 (10)	0.68799 (4)	0.0255 (3)	
C1	0.2985 (2)	0.29796 (11)	0.71599 (4)	0.0246 (3)	
C2	0.1238 (2)	0.40318 (12)	0.71220 (4)	0.0269 (4)	
C3	−0.0306 (2)	0.36569 (12)	0.66740 (5)	0.0257 (3)	
C4	−0.0433 (2)	0.15597 (12)	0.62528 (4)	0.0253 (3)	
C5	−0.2649 (2)	0.17337 (14)	0.60319 (5)	0.0331 (4)	
C6	−0.3486 (3)	0.09418 (15)	0.56701 (5)	0.0355 (4)	
C7	−0.2145 (3)	−0.00281 (14)	0.55219 (5)	0.0380 (5)	
C8	0.0064 (3)	−0.01954 (15)	0.57400 (5)	0.0374 (4)	
C9	0.0931 (2)	0.05842 (13)	0.61021 (5)	0.0297 (4)	
C10	0.5036 (2)	0.29188 (12)	0.74964 (4)	0.0243 (3)	
C11	0.5501 (2)	0.38492 (12)	0.78377 (5)	0.0276 (4)	
C12	0.7391 (2)	0.37447 (12)	0.81698 (5)	0.0282 (4)	
C13	0.8878 (2)	0.27223 (12)	0.81727 (5)	0.0272 (4)	
C14	0.8428 (2)	0.18087 (12)	0.78254 (5)	0.0289 (4)	
C15	0.6556 (2)	0.18988 (12)	0.74930 (5)	0.0266 (4)	
C16	1.0901 (3)	0.26048 (14)	0.85368 (5)	0.0342 (4)	
C17	0.0167 (2)	0.44225 (12)	0.62430 (4)	0.0247 (3)	
C18	0.2099 (2)	0.41944 (13)	0.59980 (5)	0.0302 (4)	
C19	0.2591 (2)	0.49430 (14)	0.56221 (5)	0.0317 (4)	
C20	0.1173 (3)	0.59488 (12)	0.54784 (5)	0.0294 (4)	
C21	−0.0768 (3)	0.61616 (12)	0.57187 (5)	0.0314 (4)	
C22	−0.1273 (2)	0.54094 (12)	0.60961 (5)	0.0285 (4)	
C23	0.1766 (3)	0.67715 (14)	0.50757 (5)	0.0388 (4)	
H2A	0.19920	0.48500	0.70820	0.0320*	
H2B	0.03430	0.40650	0.74020	0.0320*	
H3A	−0.19730	0.37210	0.67330	0.0310*	
H5A	−0.35870	0.23960	0.61290	0.0400*	
H6A	−0.49980	0.10670	0.55220	0.0430*	
H7A	−0.27280	−0.05700	0.52750	0.0460*	
H8A	0.09980	−0.08560	0.56390	0.0450*	
H9A	0.24470	0.04570	0.62480	0.0360*	
H11A	0.45170	0.45570	0.78420	0.0330*	
H12A	0.76760	0.43840	0.83990	0.0340*	
H14A	0.94320	0.11100	0.78180	0.0350*	
H15A	0.62960	0.12660	0.72600	0.0320*	
H16A	1.04610	0.29080	0.88410	0.0510*	
H16B	1.21930	0.31040	0.84400	0.0510*	
H16C	1.13670	0.17260	0.85660	0.0510*	
H18A	0.30930	0.35160	0.60900	0.0360*	
H19A	0.39150	0.47680	0.54600	0.0380*	
H21A	−0.17720	0.68340	0.56240	0.0380*	
H22A	−0.26150	0.55730	0.62540	0.0340*	
H23A	0.05160	0.73740	0.50010	0.0580*	
H23B	0.19620	0.62540	0.47980	0.0580*	
H23C	0.32050	0.72220	0.51660	0.0580*	

### Atomic displacement parameters (Å^2^)

**Table:**

	*U*^11^	*U*^22^	*U*^33^	*U*^12^	*U*^13^	*U*^23^
N1	0.0281 (6)	0.0256 (5)	0.0276 (6)	0.0039 (4)	−0.0019 (4)	0.0005 (4)
N2	0.0263 (5)	0.0264 (5)	0.0234 (5)	0.0016 (4)	−0.0005 (4)	0.0017 (4)
C1	0.0293 (6)	0.0236 (6)	0.0213 (6)	0.0016 (5)	0.0049 (5)	0.0019 (4)
C2	0.0328 (7)	0.0254 (6)	0.0229 (6)	0.0042 (5)	0.0047 (5)	0.0007 (5)
C3	0.0249 (6)	0.0254 (6)	0.0270 (6)	0.0035 (5)	0.0043 (5)	0.0009 (5)
C4	0.0276 (6)	0.0256 (6)	0.0228 (6)	−0.0036 (5)	0.0028 (5)	0.0033 (5)
C5	0.0301 (7)	0.0363 (7)	0.0325 (7)	0.0025 (5)	0.0007 (5)	0.0029 (6)
C6	0.0296 (7)	0.0454 (8)	0.0301 (7)	−0.0057 (6)	−0.0047 (6)	0.0062 (6)
C7	0.0461 (9)	0.0390 (8)	0.0277 (7)	−0.0096 (6)	−0.0038 (6)	−0.0025 (6)
C8	0.0430 (8)	0.0361 (7)	0.0326 (7)	0.0022 (6)	0.0003 (6)	−0.0061 (6)
C9	0.0286 (7)	0.0318 (7)	0.0282 (7)	0.0003 (5)	−0.0002 (5)	−0.0008 (5)
C10	0.0283 (6)	0.0243 (6)	0.0206 (6)	−0.0009 (5)	0.0036 (5)	0.0032 (5)
C11	0.0341 (7)	0.0242 (6)	0.0247 (6)	0.0025 (5)	0.0040 (5)	0.0012 (5)
C12	0.0366 (7)	0.0266 (6)	0.0213 (6)	−0.0036 (5)	0.0028 (5)	−0.0014 (5)
C13	0.0292 (7)	0.0286 (6)	0.0237 (6)	−0.0043 (5)	0.0019 (5)	0.0050 (5)
C14	0.0299 (7)	0.0250 (6)	0.0316 (7)	0.0025 (5)	0.0013 (5)	0.0023 (5)
C15	0.0315 (7)	0.0229 (6)	0.0252 (6)	−0.0009 (5)	0.0020 (5)	−0.0011 (5)
C16	0.0350 (7)	0.0342 (7)	0.0320 (7)	−0.0050 (6)	−0.0044 (6)	0.0026 (6)
C17	0.0247 (6)	0.0267 (6)	0.0224 (6)	0.0011 (5)	0.0000 (5)	−0.0009 (5)
C18	0.0271 (7)	0.0344 (7)	0.0292 (7)	0.0067 (5)	0.0027 (5)	0.0020 (5)
C19	0.0290 (7)	0.0390 (7)	0.0277 (7)	−0.0002 (5)	0.0060 (5)	−0.0015 (5)
C20	0.0377 (7)	0.0288 (6)	0.0210 (6)	−0.0067 (5)	−0.0018 (5)	−0.0020 (5)
C21	0.0393 (8)	0.0260 (6)	0.0282 (7)	0.0052 (5)	−0.0017 (6)	0.0008 (5)
C22	0.0292 (7)	0.0291 (6)	0.0272 (6)	0.0051 (5)	0.0031 (5)	−0.0018 (5)
C23	0.0546 (9)	0.0354 (7)	0.0264 (7)	−0.0087 (6)	0.0034 (6)	0.0017 (6)

### Geometric parameters (Å, °)

**Table:**

N1—N2	1.3758 (16)	C20—C21	1.388 (2)
N1—C3	1.4794 (17)	C20—C23	1.508 (2)
N1—C4	1.3916 (16)	C21—C22	1.393 (2)
N2—C1	1.2901 (16)	C2—H2A	0.9900
C1—C2	1.5132 (17)	C2—H2B	0.9900
C1—C10	1.4639 (16)	C3—H3A	1.0000
C2—C3	1.5464 (18)	C5—H5A	0.9500
C3—C17	1.5191 (18)	C6—H6A	0.9500
C4—C5	1.3955 (17)	C7—H7A	0.9500
C4—C9	1.3999 (18)	C8—H8A	0.9500
C5—C6	1.388 (2)	C9—H9A	0.9500
C6—C7	1.385 (2)	C11—H11A	0.9500
C7—C8	1.388 (2)	C12—H12A	0.9500
C8—C9	1.386 (2)	C14—H14A	0.9500
C10—C11	1.4001 (18)	C15—H15A	0.9500
C10—C15	1.4043 (18)	C16—H16A	0.9800
C11—C12	1.3896 (18)	C16—H16B	0.9800
C12—C13	1.3934 (18)	C16—H16C	0.9800
C13—C14	1.3980 (19)	C18—H18A	0.9500
C13—C16	1.503 (2)	C19—H19A	0.9500
C14—C15	1.3813 (18)	C21—H21A	0.9500
C17—C18	1.3935 (17)	C22—H22A	0.9500
C17—C22	1.3887 (18)	C23—H23A	0.9800
C18—C19	1.385 (2)	C23—H23B	0.9800
C19—C20	1.395 (2)	C23—H23C	0.9800
			
N2—N1—C3	112.10 (10)	H2A—C2—H2B	109.00
N2—N1—C4	119.35 (10)	N1—C3—H3A	110.00
C3—N1—C4	124.50 (11)	C2—C3—H3A	110.00
N1—N2—C1	109.02 (10)	C17—C3—H3A	110.00
N2—C1—C2	112.98 (10)	C4—C5—H5A	120.00
N2—C1—C10	121.23 (11)	C6—C5—H5A	120.00
C2—C1—C10	125.64 (10)	C5—C6—H6A	120.00
C1—C2—C3	101.74 (10)	C7—C6—H6A	120.00
N1—C3—C2	100.76 (10)	C6—C7—H7A	121.00
N1—C3—C17	112.15 (11)	C8—C7—H7A	121.00
C2—C3—C17	113.12 (10)	C7—C8—H8A	119.00
N1—C4—C5	119.68 (12)	C9—C8—H8A	119.00
N1—C4—C9	121.18 (11)	C4—C9—H9A	120.00
C5—C4—C9	119.11 (12)	C8—C9—H9A	120.00
C4—C5—C6	120.19 (13)	C10—C11—H11A	120.00
C5—C6—C7	120.84 (15)	C12—C11—H11A	120.00
C6—C7—C8	118.92 (14)	C11—C12—H12A	119.00
C7—C8—C9	121.14 (14)	C13—C12—H12A	119.00
C4—C9—C8	119.81 (12)	C13—C14—H14A	119.00
C1—C10—C11	121.26 (11)	C15—C14—H14A	119.00
C1—C10—C15	120.46 (11)	C10—C15—H15A	120.00
C11—C10—C15	118.25 (11)	C14—C15—H15A	120.00
C10—C11—C12	120.52 (11)	C13—C16—H16A	109.00
C11—C12—C13	121.35 (12)	C13—C16—H16B	109.00
C12—C13—C14	117.81 (12)	C13—C16—H16C	109.00
C12—C13—C16	121.15 (12)	H16A—C16—H16B	109.00
C14—C13—C16	121.04 (11)	H16A—C16—H16C	110.00
C13—C14—C15	121.52 (12)	H16B—C16—H16C	109.00
C10—C15—C14	120.53 (12)	C17—C18—H18A	120.00
C3—C17—C18	121.29 (11)	C19—C18—H18A	120.00
C3—C17—C22	120.40 (11)	C18—C19—H19A	119.00
C18—C17—C22	118.25 (12)	C20—C19—H19A	120.00
C17—C18—C19	120.94 (12)	C20—C21—H21A	119.00
C18—C19—C20	121.05 (12)	C22—C21—H21A	119.00
C19—C20—C21	117.84 (13)	C17—C22—H22A	120.00
C19—C20—C23	120.31 (14)	C21—C22—H22A	120.00
C21—C20—C23	121.85 (13)	C20—C23—H23A	110.00
C20—C21—C22	121.30 (13)	C20—C23—H23B	109.00
C17—C22—C21	120.61 (12)	C20—C23—H23C	110.00
C1—C2—H2A	111.00	H23A—C23—H23B	109.00
C1—C2—H2B	111.00	H23A—C23—H23C	109.00
C3—C2—H2A	111.00	H23B—C23—H23C	109.00
C3—C2—H2B	111.00		
			
C3—N1—N2—C1	12.24 (14)	N1—C4—C9—C8	177.64 (13)
C4—N1—N2—C1	170.61 (11)	C5—C4—C9—C8	−0.4 (2)
N2—N1—C3—C2	−18.27 (13)	C4—C5—C6—C7	−0.1 (2)
N2—N1—C3—C17	102.32 (12)	C5—C6—C7—C8	−0.3 (2)
C4—N1—C3—C2	−175.32 (11)	C6—C7—C8—C9	0.3 (2)
C4—N1—C3—C17	−54.74 (15)	C7—C8—C9—C4	0.0 (2)
N2—N1—C4—C5	172.10 (12)	C1—C10—C11—C12	176.68 (12)
N2—N1—C4—C9	−5.93 (18)	C15—C10—C11—C12	−1.44 (19)
C3—N1—C4—C5	−32.39 (18)	C1—C10—C15—C14	−176.72 (12)
C3—N1—C4—C9	149.59 (12)	C11—C10—C15—C14	1.41 (19)
N1—N2—C1—C2	0.12 (14)	C10—C11—C12—C13	0.3 (2)
N1—N2—C1—C10	175.95 (10)	C11—C12—C13—C14	0.90 (19)
N2—C1—C2—C3	−11.25 (13)	C11—C12—C13—C16	−179.14 (13)
C10—C1—C2—C3	173.14 (11)	C12—C13—C14—C15	−0.93 (19)
N2—C1—C10—C11	−172.36 (12)	C16—C13—C14—C15	179.11 (13)
N2—C1—C10—C15	5.72 (18)	C13—C14—C15—C10	−0.2 (2)
C2—C1—C10—C11	2.91 (18)	C3—C17—C18—C19	−176.21 (12)
C2—C1—C10—C15	−179.01 (11)	C22—C17—C18—C19	0.94 (19)
C1—C2—C3—N1	16.33 (11)	C3—C17—C22—C21	176.07 (12)
C1—C2—C3—C17	−103.56 (11)	C18—C17—C22—C21	−1.10 (19)
N1—C3—C17—C18	−36.48 (16)	C17—C18—C19—C20	0.2 (2)
N1—C3—C17—C22	146.44 (12)	C18—C19—C20—C21	−1.1 (2)
C2—C3—C17—C18	76.66 (15)	C18—C19—C20—C23	178.58 (13)
C2—C3—C17—C22	−100.43 (13)	C19—C20—C21—C22	1.0 (2)
N1—C4—C5—C6	−177.62 (13)	C23—C20—C21—C22	−178.74 (13)
C9—C4—C5—C6	0.5 (2)	C20—C21—C22—C17	0.2 (2)

### Hydrogen-bond geometry (Å, °)

**Table:**

Cg2, Cg3 and Cg4 are the centroids of the C4–C9, C10–C15 and C17–C22 rings, respectively.

**Table:**

*D*—H···*A*	*D*—H	H···*A*	*D*···*A*	*D*—H···*A*
C2—H2B···Cg3^i^	0.99	2.74	3.5766 (13)	142
C12—H12A···Cg2^ii^	0.95	2.69	3.5485 (15)	150
C16—H16C···Cg4^iii^	0.98	2.81	3.6144 (17)	140
C23—H23B···Cg4^iv^	0.98	2.77	3.5742 (16)	140

Symmetry codes: (i) *x*−1, *y*, *z*; (ii) −*x*+1/2, *y*+1/2, −*z*+3/2; (iii) −*x*+3/2, *y*−1/2, −*z*+3/2; (iv) −*x*, −*y*+1, −*z*+1.

## References

[bb1] Farrugia, L. J. (1997). *J. Appl. Cryst.* **30**, 565.

[bb2] Farrugia, L. J. (1999). *J. Appl. Cryst.* **32**, 837–838.

[bb3] Jasinski, J. P., Pek, A. E., Samshuddin, S., Narayana, B. & Yathirajan, H. S. (2010). *Acta Cryst.* E**66**, o1950–o1951. 10.1107/S1600536810025584 PMC3007262 21588275

[bb4] Oxford Diffraction (2007). *CrysAlis PRO* and *CrysAlis RED* Oxford Diffraction Ltd, Abingdon, England.

[bb5] Samshuddin, S., Narayana, B., Yathirajan, H. S., Safwan, A. P. & Tiekink, E. R. T. (2010). *Acta Cryst.* E**66**, o1279–o1280. 10.1107/S1600536810015795 PMC2979444 21579379

[bb6] Sheldrick, G. M. (2008). *Acta Cryst.* A**64**, 112–122. 10.1107/S0108767307043930 18156677

[bb7] Spek, A. L. (2009). *Acta Cryst.* D**65**, 148–155. 10.1107/S090744490804362X PMC2631630 19171970

